# Job satisfaction and work stress among physicians in Norway and Germany—A cross-sectional study

**DOI:** 10.1371/journal.pone.0296703

**Published:** 2024-01-05

**Authors:** Edgar Voltmer, Judith Rosta, Susen Köslich-Strumann, Katja Goetz

**Affiliations:** 1 Institute of Social Medicine and Epidemiology, University of Lübeck, Lübeck, Germany; 2 Institute for Studies of the Medical Profession, Oslo, Norway; 3 Institute of Family Medicine, University Medical Centre Schleswig-Holstein, Lübeck, Germany; University of Valencia: Universitat de Valencia, SPAIN

## Abstract

**Purpose:**

Physicians’ health and wellbeing are important albeit often neglected quality indicators of health care systems. The aims of the study were to compare job satisfaction and work stress among doctors in Germany and Norway, and to identify predictors for job satisfaction.

**Methods:**

All active physicians in Schleswig-Holstein, Germany (N = 13,304) and a nationwide sample of Norwegian physicians (N = 2,316) were surveyed in a cross-sectional design in 2021. Response rates of German and Norwegian physicians were n = 4,385 (33%) and n = 1,639 (70.8%), respectively. In addition to age, sex, and work-hours, the main outcome measures were the validated Job Satisfaction Scale (JSS) and the short form of the Effort-Reward Imbalance Questionnaire (ERI).

**Results:**

There were significant differences between Norwegian and German physicians in job satisfaction but with small effect sizes. All effort scores of German physicians were significantly higher and four of seven reward scores significantly lower than for their Norwegian colleagues. The proportion of German physicians in the state of a gratification crisis was significantly higher (67%) than in their Norwegian colleagues (53%). In both countries, physicians with a gratification crisis scored significantly lower on all items of job satisfaction. There were only minor gender differences in job satisfaction and effort-reward balance. Age, effort, and reward accounted for 46% of the explained variance of job satisfaction.

**Conclusions:**

Lower job satisfaction and reward in some areas and higher perceived effort of physicians in Germany than in Norway are still in favor of Norwegian working conditions, but the differences seem to diminish. The high proportions of gratification crises in both countries warrants appropriate measures for prevention and health promotion.

## Introduction

Physicians’ health and wellbeing are important albeit often neglected quality indicators of health care systems [1]. Work stress may impair and job satisfaction may elevate not only the personal wellbeing of physicians but also their quality of patient care [2]. High workload, long working hours, infrequent feedback and not least the ongoing COVID-19 pandemic are influencing factors for physicians’ stress, depression, or burnout [3–7]. In a longitudinal comparison, US physicians’ satisfaction with work-life-balance was lower and declined compared to the general population while the proportion with burnout was higher and increased [8]. In 2020, physicians’ scores for emotional exhaustion and depersonalization improved, although not in those specialties most impacted by the COVID-19 pandemic [9]. An early review found no gender differences regarding overall job satisfaction, but women were more concerned with a perceived lack of time for relationships with patients, colleagues and family, and less satisfied with mentoring and support as well as career advancement opportunities, recognition and salary [10]. In a more recent review, there were hints that female physicians may experience lower job satisfaction and further confirmation for organizational inequalities [11]. Stress, dissatisfaction, and burnout were predictors for prescribing drugs with more side effects [12] or medical errors [13–15]. Job Stress and dissatisfaction may also be a reason for quitting medical professional work or starting anew in a foreign country [16,17]. While most of German physicians who leave for abroad choose German-speaking countries like Austria or Switzerland [18], the Scandinavian countries are also well-perceived by German physicians for their good working conditions. Higher control and participation, sufficient staffing, more teamwork, higher recognition, less extended work hours, higher flexibility in working time with less work family conflict and less emotional exhaustion are examples described as distinctive for Scandinavian countries [19–23]. A former comparison of Norwegian and German physicians working in hospitals revealed a significantly higher life and job satisfaction in Norwegian physicians compared to their German colleagues [23]. This also holds true for physicians in private practice [24]. However, there have been health care reforms in those countries as well and the impact has been received critically. Healthcare reforms in Norway have addressed coordination of care between municipalities and hospitals (2012), quality and patient safety (2013), and extension of patient choices regarding treatments and providers (2014), and included a National Health and Hospital Plan 2016–2019 with goals of empowerment of patients, prioritizing mental health services, improving provision of health care and coordination between hospitals as well as strengthening of outpatient care. “Competence Shift 2020” to adapt education and training in primary care (2015), and “Live your whole life reform” to improve quality of care for older people (2017) were further reform agendas [25]. Studies from Norway revealed a perception of increasing work hours, variety of tasks and work demands in GPs [26]. This was also seen in hospital doctors who also felt an increasing economic pressure in medical decisions [27]. Consequently, decreasing levels of work satisfaction of Norwegian physicians have been reported [28]. In Germany, health care reforms were implemented in 2011 and 2015–2017 addressing general financing but also remuneration of general practitioners (2015), second opinions, specialist appointments for patients within one week with a waiting time of less than four weeks, structural funds to support the conversion of surplus hospitals (2016), consideration of persons with cognitive impairments in the new assessment procedure for need of care, and inpatient-equivalent psychiatric treatment in the home environment for psychiatric and psychosomatic patients (2017) [29]. Efforts to reduce costs due to rapid medical advances and an aging population have led to a sense of increasing administrative work and economic pressure in medical decision-making also among German physicians [30]. Despite former comparisons we therefore believe that an actual evaluation of job satisfaction and job stress in Norwegian and German physicians would be of interest for physicians and administrators.

The model of effort-reward-imbalance (ERI) introduced by Siegrist et al. [31] described work stress as an unfortunate relation between high effort and low reward and termed it a “gratification crisis” [32], which was correlated to low job satisfaction but also to poor mental health and wellbeing [33,34] as well as depression and burnout [35,36]. We chose this model because it provides a clear theoretical and methodological framework for the operationalization of work stress. It depicts relevant work conditions which are also highly significant for physicians. As such, quite a number of studies in the medical context allow a comparative perspective [37,38]. In recent studies of young physicians in Germany high proportions of up to 86% with a gratification crisis were reported [39,40]. In a longitudinal study of physicians in Norway with data from 2010 to 2019 there was a significant increase in effort and a decrease in reward in GPs but not in physicians working in hospitals [41]. There are contradictory findings about gender differences in ERI scores. No significant differences were reported for surgical residents and physicians in private practice [24,42]. On the other hand, work-family conflicts and gratification crises were highly associated with burnout in six year residents especially for women [36]. There are few studies on the development of ERI in different age groups. However, previous studies reported lower stress and higher job satisfaction in older than younger age groups of physicians [43].

Recent surveys on job satisfaction and effort-reward imbalance in Germany were restricted to groups of younger [39] or employed physicians [44] without reference to other countries. The aim of this study therefore was to compare job satisfaction and work stress in a sample of all active physicians in a northern state of Germany (Schleswig-Holstein) to a representative sample of Norwegian physicians. The research questions were: a) How do physicians in Germany and Norway differ regarding job satisfaction and job stress? b) Which predictors could be identified for job satisfaction?

## Methods

### Study design and sample description

For the German sample, in 2021 all active physicians in a northern state of Germany (Schleswig-Holstein; N = 13,303) were invited via the Medical Association of Schleswig-Holstein to participate on the survey. This included physicians working in hospitals, private practice, and public health services. Questionnaires were sent via e-mail. Reminders were sent after four and eight weeks.

Data of the Norwegian sample were drawn from the latest survey (2021) of an ongoing series of studies that survey a representative sample of Norwegian doctors every second year since 1994 with postal questionnaires addressing physician’s job satisfaction, health, and working conditions [45,46]. If respondents of the unbalanced sample leave the panel due to retirement, death, or other reasons they are replaced by younger doctors to maintain the representative nature of the sample [46,47]. In 2021, N = 2,316 physicians were addressed by the Norwegian Institute for Studies of the Medical Profession via postal questionnaires (including two reminders) that could be answered both by e-mail and by post. This sample also comprised physicians working in hospitals, private practice, and public health services.

No incentives were given in either country. In the analysis, physicians with less than 2 hours of working time, younger than 25, and older than 80 were excluded to reduce bias in terms of true active/inactive physicians (no students, no pensioners).

### Ethical considerations

Germany: This study was conducted in accordance with the guidelines provided by the Declaration of Helsinki. The study protocol was approved by the Ethics Committee of the University of Lübeck (file reference: 20–136). In a letter accompanying the questionnaire, potential participants were informed about the background and objectives of the study and the voluntary nature of participation. Participation in the survey was then considered as implied informed consent.

Datasets used in the present study are not publicly available due to strict German data protection regulations and the guidelines of the ethics committee of Lübeck University (https://www.uni-luebeck.de/forschung/kommissionen/ethikkommission.html; email: ethikkommission@uni-luebeck.de). The data contain sensitive information about the participants´ mental health and the participants have not given their consent to make the datasets publicly available. However, data are available on reasonable request from the Research Group “Health Promotion in Study and Work” at the Institute for Social Medicine and Epidemiology, University of Lübeck (www.lust.uni-luebeck.de/home.html; email: gesundstudieren@uni-luebeck.de).

Norway: This study involves human participants and was approved. According to the Regional Committee for Medical Research Ethics, the study based on “Norwegian Physician Survey—A biennial prospective questionnaire survey of a representative sample of Norwegian physicians” is exempt from review in Norway, cf. §§ 4 of The Act. The project can be implemented without approval by the Regional Committee for Medical Research Ethics (IRB 0000 1870). In addition, approval for data protection of the biennial prospective survey among Norwegian doctors was obtained from the Norwegian Social Science Data Service (Reference 19521). Participants gave informed consent to participate in the study before taking part. It was explained that participation was voluntary and that the data would be handled confidentially. The Norwegian datasets in the present study are not publicly available due strict data protection regulations and guidelines of the "Regional Committee for Medical and Health Research Ethics" (https://www.forskningsetikk.no/en/about-us/our-committees-and-commission/rek/; email: post@forskningsetikk.no) The data contain sensitive information about the participants´ mental health and the participants have not given their consent to make the datasets publicly available. However, aggregated data are available on reasonable request from "The Institute for Studies of the Medical Profession" (www.lefo.no; email: lefo@lefo.no) for researchers who meet the criteria for access to confidential data.

### Measures

Job satisfaction was measured in both countries with the ten items of the Job Satisfaction Scale (JSS) [48]. Each item is rated on a seven-point Likert scale from 1, extremely dissatisfied, to 7, extremely satisfied. The mean score is calculated without any weighting.

For work stress we used a short form of the effort-reward questionnaire (ERI) [49]. It comprises three items for the effort scale and seven items for the reward scale. While effort comprises aspects like time pressure, work interruptions and increasing demands, reward is not only estimated by income but also by opportunities for professional development, job security as well as appreciation and respect by supervisors [50]. Estimations were given on a four-point Likert scale ranging from 1, strongly disagree, to 4, strongly agree. After recoding three items of the reward scale, high scores of both scales indicated high perceived effort and reward. To identify physicians in a state of a “gratification crisis” a ratio of effort (as enumerator) and reward (as denominator) adjusted for number of items (number of reward items/number of effort items) was calculated [50]. According to this model, a ratio greater than 1.0 indicated a critically high level of work-related stress. For both measures country specific language versions were available. Sociodemographic variables were age, sex, and work-hours.

### Statistical analyses

Data analyses were conducted with SPSS for windows Version 22.0 (SPSS Inc., Chicago, IL, USA). We report univariate statistics as means and standard deviations for continuous variables and percentages for categorical variables. For categorical variables, data were analyzed using χ^2^ tests; for continuous variables, we used analyses of variance in a general linear model. Analyses of variance were adjusted for age and gender. For the ANOVA in the General Linear Model (GLM) we used partial eta^2^ as measure of effect size (according to Cohen [51]: .01 (small effect), .06 (medium effect), .14 (large effect)). The association of demography (age, sex, country), work-factors (working-time), and work stress (effort, reward scores) as independent variables with physicians’ satisfaction (mean score of all items of JSS) as the dependent variable were analysed with forced entry linear regression models with cut off scores of p < 0.05 for inclusion and p > 0.10 for exclusion.

## Results

The total response rate of German physicians was n = 4,385 (33%; not all questionnaires were fully completed, relevant numbers are given with each analysis). There was no significant difference in age between responders (mean 47.7, SD 11.5) and the total group of addressed physicians (mean age 47.0, (SD 12.2) years), but a higher percentage of female physicians (60% vs. 50% in addressed physicians) participated (p < 0.01).

For the Norwegian physicians the response was 70.8% (n = 1,639 of 2,316 addressed physicians). For both groups we included all physicians with a working time > 1 h/week as active.

Table 1 displays the sample characteristics. There was a difference in mean age between Germany (47.7, SD 11.5) and Norway (44.8, SD 12.8; p < 0.001). According to the standard residuals the differences were especially due to groups of younger physicians aged 25–34 years and the age group > 65, and a smaller group of physicians aged 50–59 years in the Norwegian sample. A greater proportion of women also responded in Norway, but the proportion between males (45%) and females (55%) was more balanced (Germany, 40% male, 60% female). The majority of Norwegian physicians (57%) worked 40–49 h/week. In Germany that statistic was 29%, but 27% worked between 50 and 59 h/week and 15% 60–79 h/week.

**Table 1:** Sample characteristics of German and Norwegian physicians.

	Germany		Norway		
	No.	%	No.	%	p_GER, NOR_
Female	1782	60.0	842	54.6	< 0.01
Male	1185	39.9	699	45.3	
Divers	2	0.1	2	0.1	
Age (Mean, SD)	47.7 (11.5)		44.8 (12.8)		< 0.01
- 34	473	16.0	438	28.4	< 0.01
35–39	346	11.7	243	15.7	
40–49	755	25.5	338	21.9	
50–59	886	29.9	235	15.2	
60–65	381	12.9	152	9.9	
> 65	120	4.1	137	8.9	
Working hours/week (Mean, SD)	45.3 (13.4)		44.7 (9.6)		n.s.
-19	71	2.2	34	2.2	< 0.01
20–29	267	8.3	32	2.1	
30–39	539	16.7	172	11.1	
40–49	942	29.1	881	57.0	
50–59	864	26.7	308	19.9	
60–79	497	15.4	114	7.4	
80-	53	2.3	4	0.3	

### Job satisfaction

There were only minor differences in the mean scores of the ten items of job satisfaction between Norwegian and German doctors (Table 2). Even if they were statistically significant, the effect sizes were (very) small (overall satisfaction with work, part. eta^2^ = 0.02; opportunities to use abilities = 0.01; variation in work = 0.01). Adjustment for age and sex only resulted in minor variations. There were also minor differences in some of JSS mean scores between male and female physicians in both countries and differences between female or male physicians between Norway and Germany, but again the effect sizes were very small. Descriptively, job satisfaction increased with age in both countries. In Norway from a mean 4.8 (SD 1.0) in the 25–34 age group to 5.3 (0.8) in the 60–65 age group and 5.5 (0.9) in the 66–80 age group. In German physicians there was an increase from a mean of 4.6 (SD 1.1) in the 25–34 age group to 5.2 (1.0) in the 60–65 age group and 5.4 (0.9) in the 66–80 age group. Though the total models and most of the differences between age groups in the post hoc test reached the significance level, the effect sizes were small.

**Table 2:** Differences between German and Norwegian physicians in the Job Satisfaction Scale (JSS).

Satisfaction with…	GER (n = 2,891) M (SD)	NOR (n = 1,515) M (SD)	p	part. eta^2^*
1. Amount of responsibility given	5.0 (1.4)	5.0 (1.5)	0.68	0.00
2. Opportunities to use abilities	5.1 (1.4)	5.4 (1.3)	< 0.01	0.01
3. Variation in work	5.0 (1.4)	5.3 (1.3)	< 0.01	0.01
4. Freedom to choose method	4.8 (1.5)	4.9 (1.5)	0.09	0.00
5. Colleagues and fellow workers	5.6 (1.2)	5.8 (1.2)	< 0.01	0.00
6. Recognition for good work	4.6 (1.7)	4.7 (1.6)	0.22	0.00
7. Rate of pay	4.8 (1.5)	4.7 (1.6)	0.01	0.00
8. Work hours	4.3 (1.7)	4.3 (1.7)	0.88	0.00
9. Physical working conditions	4.7 (1.5)	4.8 (1.5)	0.06	0.00
10. Overall job satisfaction	5.1 (1.3)	5.5 (1.2)	< 0.01	0.02

*part eta^2^: according to Cohen (51): 0.01 (small effect), 0.06 (medium effect), 0.14 (large effect).

### Effort-reward imbalance and gratification crisis

German physicians scored higher than their Norwegian colleagues in all three items of the effort score. Mean differences and effect sizes were medium to small (time pressure, part. eta^2^ = 0.04; increasing job demands, part. eta^2^ = 0.03; Table 3). The greatest difference in the reward scale was seen with respect and prestige for work, with a medium effect size (part. eta^2^ = 0.1). Respect from superiors or a respective relevant person, job security, and job promotion prospects followed with small effect sizes (part. eta^2^ = 0.03; 0.01; 0.01 respectively). There was no significant difference in prospects of job development. Adjustments for age and sex did not change these results.

**Table 3:** Comparison of effort and reward between German and Norwegian physicians.

	GER n = 2,751 M (SD)	NOR n = 1,481 M (SD)	p	part eta^2^*
*Effort*				
Constant time pressure due to a heavy workload	3.2 (0.8)	2.9 (0.8)	< 0.01	0.04
Many interruptions and disturbances in my job	3.0 (0.8)	3.0 (0.8)	< 0.01	0.00
Job has become more and more demanding	3.1 (0.8)	2.8 (0.8)	< 0.01	0.03
*Reward*				
Respect from superior or a respective relevant person	2.6 (0.8)	2.9 (0.8)	< 0.01	0.03
Prospects of my further job development are poor^a^	2.6 (0.8)	2.6 (0.9)	.676	0.00
Undesirable change in work situation^a^	2.6 (0.9)	2.6 (0.9)	.044	0.00
Poor Job security^a^	3.4 (0.7)	3.2 (0.8)	< 0.01	0.01
Respect and prestige I deserve for my work	2.4 (0.8)	2.9 (0.7)	< 0.01	0.10
Job promotion prospects are adequate	2.6 (0.7)	2.8 (0.7)	< 0.01	0.01
Income is adequate	2.4 (0.8)	2.5 (0.8)	0.02	0.00

^a^recoded: high values indicate high reward.

*part eta^2^: according to Cohen (1988) 0.01 (small effect), 0.06 (medium effect), 0.14 (large effect).

**Fig 1:**
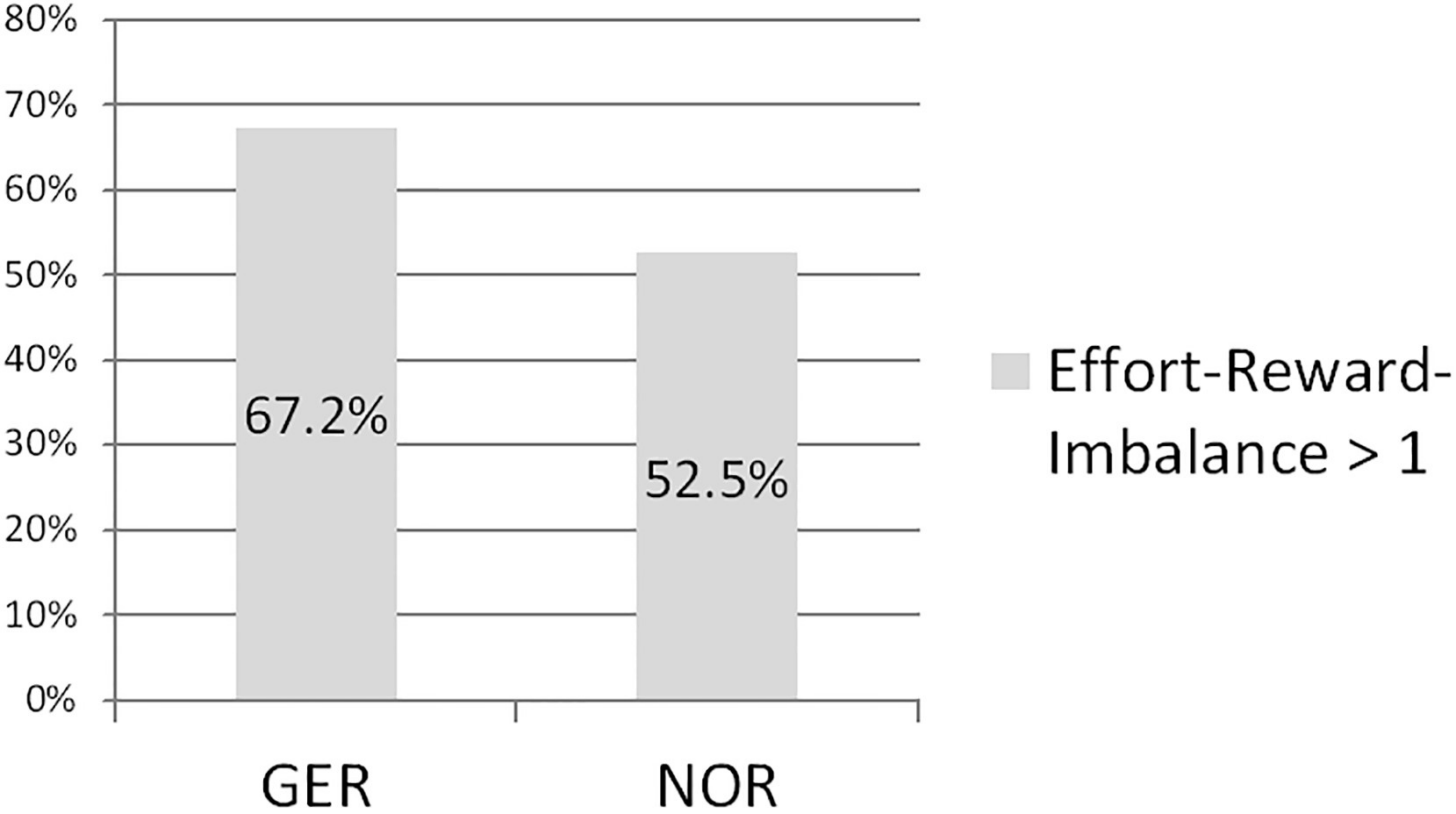
Effort Reward-Imbalance (“Gratification Crisis”) of German and Norwegian physicians.

The proportion of physicians in a state of a “gratification crisis” was significantly greater among physicians in Germany (67.2%) than in Norway (52.5%) (Fig 1).

German and Norwegian physicians with an effort-reward score > 1 scored significantly lower on all items of the job satisfaction scale in both countries (Table 4). A large effect was seen in German physicians for work hours (part. eta^2^ = 0.194) followed by medium effects for physical working conditions (part. eta^2^ = 0.146), recognition for good work (0.132), overall satisfaction (0.121), and rate of pay (0.108). In Norwegian physicians, large effects were seen for work hours (0.245) and amount of responsibility given (0.152), followed by medium effects for recognition for good work (0.135), rate of pay (0.123), and overall job satisfaction (0.119).

**Table 4:** Differences between German and Norwegian physicians with or without a gratification crisis in the Job Satisfaction Scale (JSS).

Satisfaction with…	ERI_IMBAL	GER (n = 3,228) M (SD)	p	part. eta^2^	NOR n = 1,515) M (SD)	p	part. eta^2^*
1. Amount of responsibility given	lower 1 greater 1	5.6 (1.1) 4.7 (1.5)	< 0.001	0.088	5.6 (1.2) 4.4 (1.6)	< 0.001	0.152
2. Opportunities to use abilities	lower 1 greater 1	5.6 (1.2) 4.8 (1.5)	< 0.001	0.068	5.7 (1.1) 5.2 (1.4)	< 0.001	0.042
3. Variation in work	lower 1 greater 1	5.5 (1.2) 4.8 (1.4)	< 0.001	0.049	5.6 (1.2) 5.1 (1.4)	< 0.001	0.042
4. Freedom to choose method	lower 1 greater 1	5.5 (1.2) 4.5 (1.5)	< 0.001	0.093	5.3 (1.2) 4.5 (1.5)	< 0.001	0.083
5. Colleagues and fellow workers	lower 1 greater 1	5.9 (1.1) 5.4 (1.3)	< 0.001	0.024	6.0 (1.1) 5.5 (1.3)	< 0.001	0.031
6. Recognition for good work	lower 1 greater 1	5.5 (1.2) 4.2 (1.7)	< 0.001	0.132	5.3 (1.3) 4.1 (1.6)	< 0.001	0.135
7. Rate of pay	lower 1 greater 1	5.5 (1.1) 4.4 (1.5)	< 0.001	0.108	5.2 (1.3) 4.1 (1.6)	< 0.001	0.123
8. Work hours	lower 1 greater 1	5.4 (1.2) 3.8 (1.7)	< 0.001	0.194	5.2 (1.3) 3.5 (1.5)	< 0.001	0.245
9. Physical working conditions	lower 1 greater 1	5.5 (1.1) 4.3 (1.6)	< 0.001	0.146	5.1 (1.3) 4.5 (1.6)	< 0.001	0.043
10. Overall job satisfaction	lower 1 greater 1	5.7 (1.0) 4.7 (1.4)	< 0.001	0.121	5.9 (1.0) 5.1 (1.3)	< 0.001	0.119

ERI_IMBAL: Effort-Reward Imbalance.

*part eta^2^: according to Cohen (51): 0.01 (small effect), 0.06 (medium effect), 0.14 (large effect).

There were only minor differences in the effort and reward scores between male and female physicians in the two countries (Table 5). In Norway, the effort scores were stable across the age groups from 25 to 65 with a mean of approximately 2.9 (SD 0.7) and with a small decrease in the 66–80 age group (M 2.5, SD 0.8). The same was true for the reward scale with a mean of 2.8 (SD 0.5). In German physicians the effort scores decreased from 3.3 (0.6) in the youngest age group to 3.0 (0.7) in the 60–65 age group and 2.4 (0.8) in the 66–80 age group. Reward scores increased from 2.6 (0.5) in the 25–34 age group to 2.8 (0.5) in the 60–65 age group and 3.0 (0.5) in the 66–80 age group. Again, the total models and most of the differences between age groups in the post hoc test reached the significance level, but the effect sizes were small.

**Table 5:** Effort-Reward differences between males and females in Germany and Norway.

	GER M (SD)				NOR M (SD)			
	Female (n = 1,667)	Male (n = 1,102)	p	part. eta^2^*	Female (n = 806)	Male (n = 674)	p	part. eta^2^*
*Effort*								
Constant time pressure due to a heavy workload	3.2 (0.7)	3.1 (0.8)	0.000	0.008	2.9 (0.8)	2.8 (0.9)	0.024	0.003
Many interruptions and disturbances in my job	3.0 (0.8)	3.0 (0.9)	0.041	0.003	3.0 (0.8)	2.9 (0.8)	0.050	0.002
Job has become more and more demanding	3.2 (0.8)	3.1 (0.8)	0.017	0.002	2.9 (0.8)	2.8 (0.8)	0.012	0.004
*Reward*								
Respect from superior or a respective relevant person	2.5 (0.8)	2.6 (0.8)	0.002	0.004	2.8 (0.8)	2.9 (0.8)	0.046	0.003
Prospects of my further job development are poor^a^	2.5 (0.8)	2.7 (0.9)	0.000	0.009	2.6 (0.9)	2.5 (0.9)	0.029	0.005
Undesirable change in work situation^a^	2.6 (0.8)	2.6 (0.9)	0.866	0.000	2.5 (0.9)	2.6 (0.9)	0.276	0.001
Poor Job security^a^	3.4 (0.7)	3.4 (0.7)	0.107	0.001	3.2 (0.8)	3.3 (0.8)	0.127	0.001
Respect and prestige I deserve for my work	2.3 (0.8)	2.5 (0.8)	0.000	0.011	2.9 (0.7)	2.9 (0.7)	0.046	0.002
Job promotion prospects are adequate	2.5 (0.7)	2.7 (0.7)	0.000	0.010	2.8 (0.7)	2.8 (0.7)	0.898	0.000
Income is adequate	2.4 (0.8)	2.5 (0.9)	0.000	0.007	2.4 (0.8)	2.5 (0.8)	0.040	0.004

^a^recoded: high values indicate high reward.

*part eta^2^: according to Cohen (51): 0.01 (small effect), 0.06 (medium effect), 0.14 (large effect).

Missing value analyses of JSS and ERI variables revealed very low rates in NOR of about 2%, in GER it were about 18% for JSS variables and 22% for ERI variables. MCAR Test showed that the data were not missing completely at random (p = 0.01). Extreme deviations in JSS were only seen to lower values (except work hours with no extreme deviation). The same was true for the effort variables. For the reward variables, lower, no, and higher extreme values were seen. In view of the sample size, we refrained from statistical imputation of missing values.

### Predictors of job satisfaction

In the regression analysis for the mean score with all items of job satisfaction as the dependent variable we tested age and sex (model 1), working time (model 2), effort and reward (model 3), and country (model 4) as predictor variables. In the final model age, effort, and reward were significant predictors for job satisfaction (Table 6).

**Table 6:** Predictors of job satisfaction among physicians.

Coefficients	b	SE	β	T	p	95,0% CI	
						LL	UL
(Constant)	1.933	.115		16.808	.000	1.707	2.158
Age	.011	.001	.127	10.547	.000	.009	.013
Sex	-.028	.026	-.013	-1.075	.283	-.080	.023
Working time	-.001	.001	-.007	-.514	.607	-.003	.002
Effort	-.201	.020	-.131	-10.032	.000	-.241	-.162
Reward	1.169	.024	.596	49.023	0.000	1.122	1.216
Country	.034	.026	.016	1.322	.186	-.017	.085

Job Satisfaction: F(6, 4140) = 583.1, p < 0.001; adjusted R^2^ = .457.

## Discussion

Job satisfaction and work stress are relevant issues for the health and wellbeing of health care workforce and the quality of patient care [2,52]. Scandinavian countries have been attractive for German physicians who sought better working conditions [17]. We therefore compared job satisfaction and work stress in German and Norwegian physicians and analyzed predictors for job satisfaction. There were only minor differences between countries in job satisfaction with small effect sizes. Mainly due to significantly higher scores in the effort scale, the proportion of German physicians with a gratification crisis was significantly greater than in their Norwegian colleagues. There was a strong impact of gratification crisis on job satisfaction. Age, effort, and reward accounted for 46% of the explained variance.

### Job satisfaction

In contrast to a comparison of Norwegian and German physicians working in private practice [24], in our study there were fewer significant differences in job satisfaction scores between physicians of both countries and the effect sizes were smaller. The overall job satisfaction of Norwegian physicians in our study was in line with scores reported from 2016 and 2017 [28]. Compared to a sample of Iranian physicians the satisfactions scores of the physicians in our study were much higher [53]. Given this transnational difference, dissatisfaction may be due to a relative deprivation [54] caused by upward comparison with states or physicians who are perceived as having better conditions (money, time, autonomy). Comparing job satisfaction across countries, one has to consider systemic differences on the level of health care systems in general and local working conditions. A review of studies about job satisfaction of employed physicians working in hospitals in the European Union revealed great differences [11]. The percentage of physicians who were satisfied with their work varied from 21% to 95%. Differences were particularly seen between old and new members of the EU. Health care systems of states like Lithuania, Croatia, or Hungary could be classified as the low budget–restricted access type. Increasing workload, and low income in these countries may not only have led to increased migration of physicians but also to poor job satisfaction and personal health, not least in female physicians [11,55]. Great differences have also been reported for other health care personnel like nurses in Europe and the USA, including Norway and Germany [56]. While about 37% of German nurses were dissatisfied with their job it was only 21% in Norway. Differences in work environment including staffing in terms of the patient to nurse ratio were significant influencing factors [56].

### Effort-reward imbalance and gratification crisis

While in the study of physicians in private practice a threefold proportion of German physicians presented with scores of a gratification crisis compared to their Norwegian colleagues [24], in our study the relative difference has diminished. In both countries (though measured with a different version of the ERI) the proportion of physicians with a gratification crisis has increased. The high proportion of physicians with a gratification crisis is supported by and was even higher in other studies. About 83% of young German physicians in training presented with an effort-reward ratio > 1 (the cut-off score for a gratification crisis), for a quarter it was even > 2 [40]. In subgroup analyses proportions of 82% for residents in gynecology [57] and 86% in urology residents [39] were reported. In General Practitioners with duties in Out-Of-Hours Care it was 95% [58]. In Norwegian physicians an increase from 10% to 40% was seen in GPs from 2010 to 2019, respectively, with ERI scores indicative of a gratification crisis. The proportion of hospital doctors with a gratification crisis slightly increased from 23% in 2010 to 27% in 2016 and 2019 [41]. In contrast, substantially lower proportions were reported in a study on university personnel [including physicians; 59].

In both study groups the satisfaction with work hours was the lowest of all of the items. In line with this, the significant differences between physicians with and without a gratification crisis showed the greatest difference and a large effect size for this item. However, it is important to notice that real working time was not a significant predictor of job satisfaction. Obviously the “felt” working time is more relevant than the real work hours. In our study the largest proportion of Norwegian physicians (57%) worked 40–49 h/week. In German physicians the distribution was shifted to higher working hours with about 43% working more than 50 h/week. A representative survey of German employed physicians in 2019 reported that 41% worked 49–59 h/week, and 22% worked 60–80 h/week. However, 90% of the respondents wished to work no more than 48h/week [44].

### Predictors of job satisfaction

A great number of studies have shown the additional impact of the COVID-19 pandemic on physicians’ job satisfaction, wellbeing, and burnout [for review see 60, 61]. However, the focus of our study was on regular work conditions, because even more studies before (and after) the pandemic have demonstrated that physicians’ wellbeing and possible burnout are often unfavorably influenced by these factors, independent of an acute crisis [62–64]. So, in addition to the severely increased challenges of physicians’ tasks not least due to the COVID-19 pandemic, factors like time pressure, economic demands, and increasing administrative workload [44,65] may also account for the high effort scores of the physicians in our study. As the regression analysis showed, effort and reward have significant influences on satisfaction with work. In particular, it is important to notice that there was a high effect size for “recognition for good work” in the distinction of physicians with and without a gratification crisis. In addition, “respect and prestige I deserve for my job” was significantly higher in Norwegian physicians. This illustrates the importance of non-monetary incentives to improve satisfaction and reduce stress. In a survey of employed physicians, only 36% rated mutual trust, respect, and loyalty with supervisors as (almost) high [66]. Consist with this, the delegates of the 126th German Physician Conference agreed on a resolution about resource-saving use of medical manpower and an appreciative treatment of physicians [67]. However, this must be acknowledged within the medical community as well.

Gender differences for job satisfaction and effort-reward balance in these physicians were small in both countries, affirming the results of former studies in surgical residents and physicians in private practice [10,24,42].

### Consequences for future research, patient care and personal health

Emigration of physicians from Germany to other countries had been a worrisome issue until 2011 with a peak of 3,410 physicians leaving Germany. Since then, there has been a continuous decrease of these numbers to only 1,910 in 2021 [68,69]. Changes in the perception of transnational working conditions reflected in the diminution of differences in perceived satisfaction and effort-reward ratio seen in our data may be one factor of influence. In Norway, previous health care legislation between 2012 and 2015 aiming at a better coordination between GPs and specialists and giving patients free choice of hospital led to a perception of high workload and considerable growth in work demand and may have been noticed by German physicians [28,70]. However, especially in the eastern parts of Germany [71,72], but also for example in Schleswig-Holstein [73], it is still hard to fill in vacancies in hospitals or practice locations. Future research should focus on the identification of factors that enhance work place attractivity for (younger) physicians in national representative samples. Since work stress and job satisfaction are also related to quality of care [74], our results also emphasize the need for interventions in Germany to foster physicians’ wellbeing, job satisfaction, and thus the quality of patient care.

This seems even more important since dissatisfaction and work stress expressed as a gratification crisis may not only lead to organizational consequences like part-time work but also cause symptoms and illnesses like burnout [36]. In employed German physicians about three-quarters felt that their working time affects their personal health and lead to work-family conflicts. About half often felt overloaded and 15% had to seek professional help [44]. In a longitudinal study on U.S. physicians compared to the general population the proportion of physicians with burnout symptoms was higher and increased from 46% in 2011 to 54% in 2014 [8]. Dissatisfaction with work or burnout increased the decision for part-time work [75]. It is of note that 11% of German physicians admitted to handling their personal health very carelessly and 62% felt that they should take more care for their personal health [44]. Organized approaches within the medical community, like the Villa Sana concept in Norway for physicians under stress or burned out [76], might also be a good model for Germany. In addition, there are various aspects, like delegation of administrative (and medical) tasks, digital support, and improved staffing, which are discussed to improve working conditions and job satisfaction, reduce work stress, and improve the quality of patient care. The efficacy and effectiveness of these measures should be approved in longitudinal studies.

### Strength and limitations

The opportunity to survey all active physicians in one federal state seemed more valuable in terms of representativity than a limited “representative” sample of all physicians in Germany, which could be questioned in terms of differences in federal/regional regulations, distribution of working places, medical specialty, etc. In addition, it is planned to develop preventive interventions for the physicians in this state on the basis of the study results in cooperation with the medical association and the physicians´ academy of this federal state. For the comparison we choose an available, carefully selected and maintained representative sample of Norwegian physicians. We therefore do not perceive these as convenience samples.

For this study we used validated instruments for the measurement of job satisfaction and work stress. The short form of the ERI used in this study was the latest version recommended by the authors of the instrument. Absolute scale scores are no longer strictly comparable, but psychometric analyses revealed no substantial differences to former versions [50]. Job satisfaction and effort-reward balance are two concepts that are related but distinct in the context of the workplace. Regression analysis revealed a substantial but not overwhelming contribution of effort and reward to the explained variance of job satisfaction. The response rate was satisfactory (33% in Germany and 70.8% in Norway), and was higher than for other surveys of the medical profession [46], but does not rule out the possibility of non-response bias. It is possible that the physicians with a particularly heavy workload and more stress were more reluctant to respond to the questionnaires giving an underestimation of work-stress level. On the other hand, physicians with high stress might to a larger degree want to express their opinion. In general, response rates in health personnel have tended to decrease over the years [77]. Possible reasons for the differences between Norway and Germany might be that the addressed German sample was much larger than the Norwegian one, which according to Cook [77] has been associated with lower response rates. The combination of postal and online survey in Norway and the awareness to be part of an ongoing longitudinal study might also have resulted in a higher response rate than the sole online and one time cross sectional survey in Germany [78,79]. Important for the higher response rate as well as the lower rate of missing values might also have been that all potential new members in Norway were invited in writing before the survey and asked whether they would agree to participate. If the invited members agreed to participate and signed a "consent form", the questionnaires were sent to them. Thus, it is conceivable that physicians in Norway were more committed to participating in the surveys and responding to the questions. With respect to the much larger study group in Germany, it must also be kept in mind that even small differences can become statistically significant. We therefore consequently added information about effect sizes, which indeed were often small. The cross-sectional study design does not allow causal interpretations. Survey data were collected in one federal state of Germany (Schleswig-Holstein); reported results may therefore be not representative of all physicians in Germany. In contrast to the German sample, the Norwegian sample represents a nation-wide selection. Another limitation is that the Norwegian sample comprises a larger proportion of younger and male doctors. However, adjusting for age and did not change the main results. Sample differences are therefore not likely to influence the reported results. Because perceived level of job satisfaction and level of work stress varies with individual characteristics such as personality and coping style [80], well-being [1,81], and mental and physical health status [82–84], it would also be important to include these co-variates in future analyses.

## Conclusions

Lower job satisfaction and reward as well as higher perceived effort of German physicians compared to their Norwegian colleagues still indicate a better working context for Norwegian physicians. However, the differences have decreased and the proportion of Norwegian physicians with gratification crises is also high. Physicians in both countries may therefore profit from active prevention and health promotion, especially with respect to effort-reward balance.
